# Nurse-led prehospital obstetric care through the lens of the Three Delays Model: an experience report

**DOI:** 10.1590/0034-7167-2025-0419

**Published:** 2026-09-28

**Authors:** Alan Messala de Aguiar Britto, Edymara Tatagiba Medina, Ricardo José Oliveira Mouta, Sandra Cristina de Souza Borges Silva

**Affiliations:** I Universidade do Estado do Rio de Janeiro. Rio de Janeiro, Rio de Janeiro, Brazil; II Universidade Federal do Estado do Rio de Janeiro, Hospital Universitário Gaffrée e Guinle. Rio de Janeiro, Rio de Janeiro, Brazil

**Keywords:** Emergency Care, Prehospital, Obstetrics, Obstetric Nursing, Maternal Mortality, Ambulance., Atención de Emergencia Prehospitalaria, Obstetricia, Enfermería Obstétrica, Mortalidad Materna, Ambulancia.

## Abstract

**Objectives:**

to report the care provided by nurses in the transportation module of the *Cegonha Carioca* Program, establishing connections with the Three Delays Model.

**Methods:**

experience report describing the care delivered by nurses to pregnant and postpartum women from 2012 to 2024, based on lived experience, public data, and the scientific literature.

**Results:**

public awareness of the ambulance service, along with the quality and humanization of care, aligns with reducing the delay in seeking care; Free access to the service, the strategic location of ambulances, and the presence of a designated referral maternity hospital facilitate the identification of and access to health services; Efficient communication, prioritization of care, and alert systems support timely and appropriate care.

**Conclusions:**

care provided in the ambulance is grounded in a humanized model and includes actions aimed at preventing delays. This modality of care is believed to represent a model of prehospital obstetric assistance capable of reducing maternal mortality.

## RESUMEN

**Objetivos:**

relatar la atención desarrollada por las enfermeras en el módulo de transporte del *Programa Cegonha Carioca*, estableciendo vínculos con el Modelo de los Tres Retrasos.

**Métodos:**

relato de experiencia que describe la atención brindada por enfermeras a gestantes y puérperas entre 2012 y 2024, basado en la vivencia profesional, en datos públicos y en la literatura científica.

**Resultados:**

la divulgación del servicio de ambulancia, junto con la calidad y la humanización del cuidado, se relacionan con la reducción del retraso en la búsqueda de atención; La gratuidad del servicio, la ubicación estratégica de las ambulancias y la existencia de una maternidad de referencia facilitan la identificación y el acceso al servicio de salu; La comunicación eficiente, la priorización del atendimiento y los sistemas de alerta favorecen la provisión de un cuidado adecuado en el momento oportuno.

**Conclusiones:**

la atención prestada en la ambulancia se basa en un modelo humanizado e incluye acciones orientadas a prevenir los retrasos. Se considera que esta modalidad de cuidado representa un modelo de atención obstétrica prehospitalaria capaz de reducir la mortalidad materna.

## INTRODUCTION

In Brazil, weaknesses in access to timely care continue to be key determinants of maternal mortality, which remains at worrying levels. The maternal mortality ratio (MMR) remained stable between 2010 and 2019 (55.31 deaths per 100,000 live births) but rose sharply during the Covid 19 pandemic (71.97 deaths per 100,000 live births), figures that are nearly twice as high as the target set for 2030 (30 deaths per 100,000 live births)^(1)^. Unfavorable obstetric and neonatal outcomes, influenced by social determinants of health and by care related factors^(1)^, can be understood through the lens of the Three Delays Model: (i) delay in deciding to seek care; (ii) delay in identifying and reaching an appropriate health service; and (iii) delay in receiving adequate care at the right time. Taken together, these delays reveal the persistence of regional and social inequalities that compromise the safety of pregnancy and childbirth in the country^(2)^.

Brazil’s national public health policies aimed at preventing maternal and neonatal morbidity and mortality include actions to reorganize and improve the quality of care to address these weaknesses. In this context, two initiatives stand out: the *Cegonha* Network (CN) and the Alyne Network (AN). The latter expands actions targeting vulnerabilities related to race and skin color, supports the expansion of Normal Birth Centers across the country, and strengthens the logistical system. Both networks are structured into components that integrate different levels of healthcare and propose actions in prenatal care, childbirth and birth, postpartum and child health, as well as logistical organization, which strengthens medical transport and user regulation. The logistical component, designed to ensure access and problem solving capacity, establishes the “*vaga sempre*” (always a bed available) rule, which guarantees hospitalization for women or newborns who are severely ill or potentially at risk, considering their linkage to the network. Additionally, inter hospital transport under safe conditions is guaranteed, taking into account the potential need for intensive care^(3,4)^.

At the time of the agreement with *Cegonha* Network, the municipality of Rio de Janeiro established the *Cegonha Carioca* (CC) Program in response to the high rates of maternal and neonatal mortality and to the frequent “pilgrimage” of pregnant women in search of care. The program enabled the organization of prenatal and childbirth care through systematized actions such as access to serological testing, improvements in the care environment, linking each woman to a reference maternity hospital according to her place of residence and obstetric risk classification, maternity visits to strengthen the bond, and the provision of an ambulance to ensure safe transport - the *Cegonha Carioca* Ambulance (CCA)^(5)^.

Considering that maternal mortality reflects problems in the organization of the women’s healthcare network and in social inequities, strengthening the logistical components of the system can reduce delays and lead to better outcomes, contributing to the reduction of maternal mortality. In this context, the transportation module of the CC in the city of Rio de Janeiro - representing the logistical system of both *Cegonha* and Alyne Networks - created the CCA, staffed by nurses and drivers, to provide humanized and qualified care aimed at reducing the pilgrimage of pregnant women and maternal and neonatal mortality^(5)^.

## OBJECTIVES

To report the care provided by nurses in the transportation module of the *Cegonha* Carioca Program, establishing connections with the Three Delays Model.

## THEORICAL FRAMEWORK

### Three Delays Model

Based on an extensive review of the literature that examined studies from the fields of health and social sciences on maternal mortality, the Three Delays Model was proposed in the early 1990s. The model asserts that most maternal deaths could be avoided if care were provided at the appropriate time, recognizes that delays are multifactorial, and classifies them into three types, which will be described below^(2)^.

The first type is the delay in deciding to seek care. Socioeconomic, cultural, personal, individual, and community factors are essential to understanding this delay. Limited financial resources and low educational attainment, the woman’s and companion’s perception of the quality of care provided by health institutions, the woman’s lack of autonomy and the influence of the companion - especially when shaped by cultural elements related to patriarchy or beliefs in alternative forms of care - lack of awareness of risk, distance, and the cost of health services are all recognized as factors contributing to this delay. It is understood that promoting health literacy, improving emergency and urgent care through professional training, ensuring care coordination, and enhancing the infrastructure of health institutions would support the decision to seek healthcare services^(2)^.

The uneven territorial distribution or absence of health facilities, the scarcity or unavailability of transportation or ambulances, the time required and the high costs of travel for women, as well as failures in referral during prenatal care, are the main factors responsible for the delay in identifying and reaching an appropriate health service (the second delay). Among the actions proposed to mitigate this delay are the construction of health facilities, the creation of effective communication systems between teams, and the implementation of a safe regulation, referral, and transport system (hospital beds and transportation)^(2)^.

The delay in receiving adequate care after accessing the health system constitutes the third delay and is associated with failures in professional decision-making (lack of referral, incorrect, imprecise, or late diagnosis, and delays in decision-making), problems in the management of material resources (shortages or absence of medications and supplies), and ineffective management of human resources (insufficient staffing, limited qualifications, and inadequate communication). To prevent this delay, it is necessary to ensure appropriate staffing and supply levels, develop and improve protocols and forms, and promote effective communication^(2)^.

Based on the Three Delays Model, maternal death is understood as a phenomenon influenced by social, economic, cultural, behavioral, biological, and institutional factors, including accessibility and the quality of care. In this sense, women’s health policies aimed at reducing maternal mortality must include actions that go beyond addressing biological aspects and that are capable of preventing delays in care. This report presents actions implemented by the CCA that seek to reduce these delays and, consequently, decrease maternal mortality.

## METHODS

This is an experience report that describes the service provided by nurses in the CCA. The experience took place between September 2012 and July 2024 in the CCA units distributed across the bases of Rio de Janeiro. It addresses the management, planning, and implementation of care practices carried out by the CCA nursing team and provided to pregnant and postpartum women who are eligible for and request the service. The report is grounded in the lived experience of an ambulance nurse and the researchers, in publicly accessible data (https://osinfo.prefeitura.rio/), and in the researchers’ expertise in obstetric care. It establishes connections between components of the care provided and the concepts underlying the Three Delays Model, offering a critical analysis of the context. It also draws on the literature on prehospital care, with a focus on obstetric services, to present the strengths and weaknesses of the program. As this is an experience report and does not involve human subjects at risk, the study did not require review by a Research Ethics Committee.

## EXPERIENCE REPORT

The transportation module of the CC began in 2011 with five ambulances that covered specific areas of the city of Rio de Janeiro. In September 2012, the service was expanded to operate with ten ambulances and to cover the entire municipality. The fleet currently consists of twelve ambulances staffed by a nurse and a driver, serving all women who completed prenatal care within the public health system and who are within the city limits at the time they request assistance.

Once prenatal care is initiated at the primary health unit, pregnant women are informed about the maternity hospital where they will give birth and about the CCA service. They receive the phone number of the municipal call center, which operates 24 hours a day and is responsible for processing their request. Additionally, to strengthen the bond with the reference maternity hospital, the pregnant woman and a companion of her choice participate in the *Visita Cegonha* during the third trimester, where they become familiar with the facility’s sectors and professionals.

Ambulance assistance may be requested by a primary health unit, a hospital unit, or directly by the user from her home. When the request comes from a health facility, the woman’s complaints and obstetric history are evaluated by the municipal regulation center, which, based on the level of complexity required, dispatches either the CC ambulance or an advanced ambulance staffed by a physician and a nurse. Home requests are made through calls to the municipal call center, which, after receiving the call, contacts the regulation center responsible for dispatching the CCA^(5,6)^.

The care provided is organized as follows: upon arriving at the health facility and being informed of the case, or upon arriving at the pregnant woman’s home, the nurse welcomes the woman and performs risk classification. To do so, the nurse uses active listening during the anamnesis, reviews the prenatal card, assesses vital signs, and conducts the obstetric examination^(5,6)^. Based on these data, risk classification is carried out using the Manchester Triage System, and in 2023 the Modified Early Obstetric Warning Score (MEOWS) was implemented. MEOWS is an early warning scale that indicates high risk for maternal morbidity or mortality, while the Manchester Triage System is a risk classification method used to determine priority of care in emergency units based on clinical condition and urgency.

After analyzing and classifying the woman’s risk, the nurse plans and implements the appropriate care. When the request comes from a health facility, the woman is transported to the hospital or maternity unit that best meets her needs, according to municipal regulation. In home based calls, when guidance and interventions are sufficient to address the woman’s needs, the nurse may advise her and her family to remain at home - an option permitted when the Manchester classification is green or blue; in all other cases, the woman is referred to the designated maternity hospital. The ambulance nurse’s consultation only ends once the pregnant woman has been received at the reference facility, ensuring continuity of humanized nursing care.

It is important to highlight that in November 2024 the process of digitalizing the service began. Records that were previously handwritten are now completed in a dedicated mobile application, stored in the cloud, and sent to the healthcare facility that will receive the woman. The goal is for the risk classification performed by the ambulance nurse to be communicated immediately to the maternity team so that the necessary resources for care can be prepared in advance.

Regarding service indicators, data collected from one of the teams showed that 692 calls were made over a four month period in 2013^(5)^, and that from 2012 to 2023 the twelve CCA units responded to a total of 174,476 requests (an average of 14,549 per year). Recent data from the transparency portal of the Rio de Janeiro City Government (available at https://osinfo.prefeitura.rio/) also show that in 2023 and 2024, 24,730 calls were made to the ambulance service, of which only 1,395 were not completed due to call center cancellation or because the woman left the primary care unit or her residence before the ambulance arrived. This corresponds to 94.4% of calls being effectively attended. Among these, 100% of care forms were fully completed and signed by the nurses, and 99.8% of users reported being satisfied or very satisfied with the service. During the same period, nearly half of all responses (48.9%) took less than 30 minutes from dispatch to arrival at the user’s location-an expressive result considering the size of the municipality (1,200.33 km^2^), its geographic complexity, and the number of ambulances available.

The care provided to women in the CCA is guided by the principles of autonomy, protection of women’s rights, resolvability, and shared care, which underpin the humanized care model. In this context, the welcoming approach and risk classification lead the nurse’s decision-making, grounded in attentive listening and clinical judgment supported by scientifically based protocols.

## DISCUSSION

Maternal mortality in Brazil remains at high levels, and various policies, programs, and actions have been developed to reduce these numbers. In Rio de Janeiro, the CC was created, whose logistical module established an ambulance service with specific characteristics. This report sought to describe the care provided by nurses in the CCA, compare it with ambulance services for pregnant women in other locations, and discuss the program by connecting its actions to the Three Delays Model, highlighting how these actions may contribute to reducing mortality.

Socioeconomic and cultural factors, as well as perceptions of the health service, are known to be key to understanding the first delay. Although it is not possible to directly modify socioeconomic conditions, strengthening the bond with prenatal care and with the reference maternity hospital, as well as presenting and reinforcing the importance of using the ambulance service, are measures that influence cultural factors and perceptions of care. Other models of maternity ambulances outside Brazil do not provide home visits; instead, they function solely as a safe means of transport between health facilities^(7,8)^. In contrast, the CCA goes to the woman’s home, provides rapid and humanized care, and is highly rated by users. In many cases, the nurse is able to address the woman’s and her family’s needs directly at home through guidance and clarification of doubts. Additionally, professionals are routinely trained and evaluated to ensure high quality service, which likely strengthens users’ trust in the health system and encourages timely care seeking.

The actions of the CCA aimed at reducing the second delay also involve other components of the CC Program. In the municipality, every woman - except those referred to high risk prenatal care - is informed by the primary care team about her designated maternity hospital, which is determined by municipal management based on proximity between the woman’s residence and the hospital. In addition, the number of ambulances and the locations from which they are dispatched were strategically planned to strengthen care in areas with the greatest service gaps and barriers to access. All these measures facilitate the identification of and arrival at the appropriate service, as they help prevent overcrowding and provide or facilitate transportation^(2)^.

Experience in African countries has shown a reduction in maternal mortality rates following the implementation of ambulances staffed by nurses or midwives who received specific training to manage obstetric emergencies^(7,8)^. In the case of the CCA, the phone number is provided to the woman during prenatal care, and assistance is only a phone call away - a low cost tool widely accessible to the population. After the ambulance nurse’s assessment, if necessary, referral to the appropriate facility is carried out free of charge and safely to the designated maternity unit. Therefore, the CCA expands upon the activities performed by African maternity ambulances and demonstrates itself to be a safe service that reduces costs for women, reinforcing its effectiveness as a strategy against the second delay^(2)^.

The third delay refers to aspects related to the quality of care provided and involves the professional’s decision making process. Decision making is more accurate when the professional is well trained. In this regard, ambulances staffed by trained midwives and nurses in The Gambia and Burundi have shown excellent results^(7,8)^, whereas prehospital care nurses often report feeling fearful and apprehensive, as childbirth is a special and relatively rare event that may involve complications they do not feel prepared to manage^(9)^. As in the African ambulance models, the CCA is staffed by trained nurses who participate in continuous professional development activities aimed at reducing decision making errors and providing appropriate care beginning in the woman’s home.

Improved and more effective communication is another aspect of the CCA designed to reduce the third delay. This type of direct communication proved effective in Burundi, where receiving prior information about the case allowed the health team to plan appropriate care^(8)^. A similar action in the CCA is the recent implementation of a tablet based application that enables direct communication between the ambulance team and the maternity unit. However, because it was implemented only recently, its impact has not yet been measured. In addition to early communication, the use of risk classification and the MEOWS system are tools that help standardize care and signal to all involved which cases require priority attention and early intervention.

It is worth noting that an ambulance service in Botucatu, São Paulo, staffed by professionals who were not specialists in obstetric care, assisted a total of 358 women with obstetric complications in 2012 and evaluated the appropriateness of these calls based on risk classification. According to the authors, only 24.4% of the calls were considered appropriate (classified as yellow, orange, or red)^(10)^. In the CCA, all calls - regardless of classification - are considered appropriate, as each represents an opportunity to provide guidance, promote accessibility, and improve the quality of care for women. Furthermore, the number of calls handled by the program shows that it is a large scale service, with volumes far exceeding those observed in other locations in Brazil^(10)^ and abroad^(7-9)^. These data reflect the importance of the service, its magnitude, and its impact on maternal and neonatal health.

The main limitation of this study is that, as an experience report with a theoretical discussion of the delays, it does not present data demonstrating the impact of the ambulance service on reducing the three delays. Even so, it is believed that the CCA helps reduce the “pilgrimage” of pregnant women and overcrowding in maternity hospitals, supports responsible transfers from primary care or maternity units to the appropriate reference facility, and expands access to healthcare (both within and outside institutions for all women in the municipality). In addition, timely care, safe transport, the use of risk classification and MEOWS, and the continuous training offered to nurses and drivers - combined with humanized care - make the ACC not only successful in its objectives but also a prehospital care service for pregnant and postpartum women grounded in humanized care, a model not yet described in the literature. It may contribute to reducing maternal and neonatal mortality by preventing the three delays and may serve as a model for other settings.

## FINAL CONSIDERATIONS

It is evident that the care provided by nurses in the transportation module of the CC Program is grounded in the humanized care model and includes actions that directly align with the Three Delays Model. It can be concluded that the ambulance nurse’s role in preventing the first delay depends partly on how well the service is communicated by the prenatal care team, but also on the quality of the care provided - especially in cases where the woman remains at home. The mere availability of the ambulance service helps prevent the second delay, and the fact that the ambulance is staffed by a highly trained nurse in obstetric care is a factor that mitigates the third delay. Additionally, risk classification, the use of MEOWS, and direct communication between the ambulance and the maternity emergency department are tools that help prevent the final delay.

Therefore, the systematically developed actions aim to prevent delays in accessing the facility that best meets the woman’s needs and in receiving timely care. Given the absence of similar experiences described in the literature, the prehospital obstetric care model developed by the CC Program can be considered pioneering in its effort to improve maternal and neonatal care and, consequently, reduce maternal mortality.

## Data Availability

The research data are available within the article.
