# Supplementary material for: Nurse-led prehospital obstetric care through the lens of the Three Delays Model: an experience report
Source: Rev Bras Enferm. 2026 Sep 28;79:e20250419. doi: 10.1590/0034-7167-2025-0419 (PMC13623389; doi:10.1590/0034-7167-2025-0419)
Supplement: Translation

# Translation: Atendimento pré-hospitalar obstétrico por enfermeiras à luz do Modelo dos Três Atrasos: relato de experiência

**DOI:** 10.1590/0034-7167-2025-0419pt

**Authors:** Alan Messala de Aguiar Britto, Edymara Tatagiba Medina, Ricardo José Oliveira Mouta, Sandra Cristina de Souza Borges Silva

**Affiliations:** I Universidade do Estado do Rio de Janeiro. Rio de Janeiro, Rio de Janeiro, Brasil; II Universidade Federal do Estado do Rio de Janeiro, Hospital Universitário Gaffrée e Guinle. Rio de Janeiro, Rio de Janeiro, Brasil

**Keywords:** Atendimento Pré-Hospitalar, Obstetrícia, Enfermagem Obstétrica, Mortalidade Materna, Ambulância.

## Abstract

**Objetivos:**

relatar a assistência desenvolvida pelas enfermeiras no módulo transporte do Programa Cegonha Carioca, estabelecendo nexos com o Modelo dos Três Atrasos.

**Métodos:**

relato de experiência que descreve a assistência de enfermeiras a gestantes e puérperas de 2012 a 2024, embasado na vivência, em dados públicos, e na literatura.

**Resultados:**

a divulgação da ambulância, a qualidade e a humanização do cuidado dialogam com a redução do atraso em buscar o cuidado; a gratuidade do serviço, localização estratégica das ambulâncias e existência de maternidade de referência facilitam a identificação e o alcance do serviço de saúde; a comunicação eficiente, priorização do atendimento e sistemas de alerta conduzem ao cuidado adequado em momento oportuno.

**Conclusões:**

a assistência prestada na ambulância é baseada no modelo humanizado e tem ações que visam prevenir os atrasos. Acredita-se que esta modalidade de cuidado represente um modelo de atendimento pré-hospitalar obstétrico capaz de reduzir a mortalidade materna.

## INTRODUÇÃO

No Brasil, as fragilidades no acesso ao cuidado oportuno continuam sendo determinantes para a mortalidade materna, que permanece em níveis preocupantes. No Brasil, a razão de mortalidade materna (RRM) manteve-se constante entre 2010 e 2019 (55,31 óbitos/100.000 nascidos vivos), mas aumentou abruptamente com pandemia da Covid-19 (71,97 óbitos/100.000 nascidos vivos), valores praticamente duas vezes maiores do que objetivo para 2030 (30 óbitos/100.000 nascidos vivos)^(1)^. Os desfechos obstétricos e neonatais desfavoráveis, impactados por determinantes sociais de saúde e por fatores assistenciais^(1)^, podem ser compreendidos à luz do modelo dos Três Atrasos: (i) atraso na decisão de buscar o cuidado; (ii) atraso em identificar e alcançar o serviço adequado; e (iii) atraso em receber o cuidado adequado no momento oportuno. Esses atrasos, combinados, revelam a persistência de desigualdades regionais e sociais que comprometem a segurança da gestação e do parto no país^(2)^.

As políticas públicas de saúde de prevenção à morbimortalidade materna e neonatal vigentes nacionalmente preveem ações de reorganização e qualificação da atenção para resolução dessas fragilidades. Assim, destaca-se a Rede Cegonha (RC) e a Rede Alyne (RA), sendo a segunda responsável por estender as ações direcionadas às vulnerabilidades relacionadas à raça/cor, ampliação dos Centros de Parto Normal no país e qualificação do sistema logístico. Ambas se organizam em componentes que integram os diferentes níveis de atenção à saúde e propõem ações no pré-natal, parto e nascimento, puerpério e atenção à saúde da criança, e na organização logística, que fortalecem o transporte sanitário e a regulação das usuárias. O componente logístico para garantir o acesso e resolutividade estabelece a regra “vaga sempre”, que garante a hospitalização da mulher ou recém-nascido grave ou potencialmente grave, considerando a sua vinculação à rede. Ademais, é garantido o transporte inter-hospitalar em condições seguras, levando-se em conta a eventual necessidade de cuidados intensivos^(3,4)^.

Na ocasião da pactuação com a RC, o município do Rio de Janeiro estabeleceu o Programa Cegonha Carioca (CC) em resposta às elevadas taxas de mortalidade materna e neonatal e à peregrinação de gestantes. Este propiciou a organização da assistência pré-natal e no parto, por meio de ações sistematizadas tais como o acesso as sorologias, melhorias na ambiência do cenário assistencial, a vinculação da mulher à maternidade de referência segundo local de moradia e classificação do risco obstétrico, a visita à maternidade para fomentar o vínculo e a disponibilização da ambulância para o transporte seguro, a Ambulância do Cegonha Carioca (ACC)^(5)^.

Considerando que a mortalidade materna revela problemas na organização da rede de atenção à mulher e as iniquidades sociais, a qualificação dos dispositivos do sistema logístico pode minimizar os atrasos e propiciar melhores desfechos, contribuindo para o controle da mortalidade materna. Neste sentido que na cidade do Rio de Janeiro o módulo transporte do CC, que representa o sistema logístico da RC e da RA, criou a ACC tripulada por enfermeiras e motoristas que propõe um cuidado humanizado e qualificado com o intuito de reduzir a peregrinação de gestantes e a mortalidade materna e neonatal^(5)^.

## OBJETIVOS

Relatar a assistência desenvolvida pelas enfermeiras no módulo transporte do Programa Cegonha Carioca, estabelecendo nexos com o Modelo dos Três Atrasos.

## REFERENCIAL TEÓRICO

### Modelo dos Três Atrasos

A partir de uma grande revisão da literatura, que buscou trabalhos da área de saúde e das ciências sociais sobre mortalidade materna, foi proposto no início da década de 1990 o Modelo dos Três Atrasos. O modelo diz que a maioria das mortes maternas seriam evitadas se fossem tratadas no momento adequado, que os atrasos são multifatoriais, e os classificou em três tipos que serão descritos à frente^(2)^.

O primeiro é o atraso em decidir buscar o cuidado. Os fatores socioeconômicos, culturais, pessoais, individuais e comunitários são essenciais para entender este atraso. Uma reduzida condição financeira e baixa escolaridade, a percepção (da mulher e acompanhante) sobre qualidade do cuidado prestado pelas instituições de saúde, a falta de autonomia da mulher e a influência do(a) acompanhante, especialmente em termos culturais (relacionados ao patriarcado e às crenças em cuidados alternativos), o desconhecimento do risco, a distância e o custo dos serviços de saúde são reconhecidos como fatores que influenciam neste atraso. Entende-se que promover o letramento em saúde, aprimorar o atendimento das urgências e emergências com treinamento profissional, garantir a coordenação do cuidado e aperfeiçoamento da infraestrutura das instituições ajudariam na decisão de buscar o serviço de saúde^(2)^.

Por outro lado, a distribuição territorial desequilibrada ou ausência das unidades de saúde, a escassez ou indisponibilidade de meios de transporte ou ambulâncias, o tempo e os elevados custos de deslocamento da mulher, bem como a falha no encaminhamento durante o pré-natal são os principais responsáveis pelo atraso em identificar e alcançar o serviço adequado (segundo atraso). Dentre as ações propostas para inibir este atraso estão a construção de unidades de saúde, criação de sistemas eficazes de comunicação entre as equipes e implementação de um sistema de regulação, encaminhamento e traslado seguros (leitos hospitalares e transporte)^(2)^.

O atraso em receber o cuidado adequado ao acessar o sistema de saúde é o terceiro atraso, e se relaciona com as falhas na tomada de decisão dos profissionais (não encaminhamento, diagnóstico errado, impreciso ou tardio e demora na decisão), com os problemas no gerenciamento de recursos materiais (escassez ou ausência de medicamentos e insumos) e com o gerenciamento ineficaz de recursos humanos (falta de dimensionamento, pouca qualificação, comunicação inadequada). Para evitar este atraso, é preciso realizar o adequado dimensionamento de pessoal e de insumos, criar e aprimorar protocolos e formulários e estimular a comunicação adequada^(2)^.

Com base no modelo dos três atrasos, nota-se que o óbito materno é um fenômeno influenciado por fatores sociais, econômicos, culturais, comportamentais, biológicos e institucionais (acessibilidade e qualidade do cuidado). Neste sentido, as políticas de saúde da mulher que visam reduzir a mortalidade materna precisam de ações que vão além de tratar o biológico, e consigam evitar os atrasos. Aqui serão relatadas ações da ACC que visam coibir os atrasos e, consequentemente, reduzir a mortalidade materna.

## MÉTODOS

Trata-se de um estudo do tipo relato de experiência que descreve o serviço prestado pelas enfermeiras na ACC. A experiência aconteceu entre setembro de 2012 a julho de 2024 nas ACC que ficam distribuídas nas bases do Rio de Janeiro. A experiência aborda o gerenciamento, o planejamento e a implementação das práticas assistenciais vivenciadas pela equipe de enfermagem da ACC, prestadas à população de gestantes e puérperas que têm direito e solicitam a prestação do serviço. É um relato alinhado à vivência no serviço sob a ótica de um enfermeiro da ambulância e dos pesquisadores, a dados de acesso público (https://osinfo.prefeitura.rio/) e à expertise dos pesquisadores sobre a assistência obstétrica. Este relato estabelece correlações entre os componentes da assistência e os conceitos que embasam o Modelo dos Três Atrasos, oferecendo uma análise crítica do cenário, além de utilizar a literatura sobre assistência pré-hospitalar com foco em atendimentos obstétricos, para apresentar pontos fortes e fracos do serviço. Por se tratar de um relato de experiência e não ter sujeitos que possam ser colocados em risco, a pesquisa não passou pelo Comitê de Ética em Pesquisa.

## RELATO DO CASO

O módulo transporte do Programa CC teve início em 2011 com cinco ambulâncias que cobriam áreas específicas da cidade do Rio de Janeiro. Em setembro de 2012 o serviço foi ampliado, passando a funcionar com 10 ambulâncias, e a cobrir todo o município. Atualmente a frota é composta por 12 ambulâncias tripuladas por uma enfermeira e um condutor e atende a todas as mulheres que realizaram o pré-natal na rede pública e que estejam dentro dos limites do município quando solicitarem o atendimento.

Uma vez iniciado o pré-natal na unidade básica de saúde, as gestantes são informadas sobre a maternidade onde irá parir e sobre o serviço da ACC. Elas recebem o contato telefônico da central da prefeitura que funciona 24 horas por dia e é responsável por dar seguimento ao chamado. Além disso, para promover o vínculo com a maternidade de referência, a gestante e um acompanhante de sua escolha fazem a Visita Cegonha no terceiro trimestre da gestação, onde conhecem setores e profissionais da instituição onde irão parir.

O atendimento da ambulância pode ser solicitado pela unidade básica de saúde (UBS), por uma unidade hospitalar ou pela usuária no seu domicílio. A partir da solicitação feita pelas unidades de saúde, as queixas e informações do histórico obstétrico da mulher são avaliadas pela regulação municipal, que pelo nível de complexidade do cuidado demandado mobiliza ou a ambulância do Cegonha Carioca ou uma ambulância avançada (tripulada com médico e enfermeira). Os chamados domiciliares são feitos através de ligações para a central da prefeitura que, após receber a ligação, contacta a central de regulação, responsável por disparar a ACC^(5,6)^.

Os atendimentos são sistematizados da seguinte forma: ao chegar para o atendimento na unidade de saúde e ser informado do caso, ou chegar no domicílio da gestante, a enfermeira acolhe a mulher e realiza a classificação de risco. Para isso, a profissional utiliza a escuta ativa na realização da anamnese, procede a leitura do cartão pré-natal, avaliação de sinais vitais e o exame obstétrico da usuária^(5,6)^. A partir destes dados é realizada a Classificação Risco pelo Protocolo de Manchester e, em 2023, foi implementado o Sistema de Alerta Obstétrico Precoce (MEOWS - *Modified Early Obstetric Warning Score*). O MEOWS é uma escala de alerta precoce que indica alto risco para morbidade ou mortalidade materna, e o Protocolo de Manchester é um método de classificação de riscos utilizado para determinar quais são os cuidados prioritários nas unidades de emergência com base em sua condição clínica e na urgência por atendimento.

Após a análise e classificação do risco da mulher, a enfermeira planeja e implementa seu cuidado. Nos atendimentos solicitados por unidades de saúde, a mulher é transportada até o hospital ou maternidade adequados às suas necessidades, segundo orientação da regulação municipal. Nos atendimentos residenciais, quando as orientações e intervenções forem capazes de sanar as demandas apresentadas, a enfermeira pode orientar a mulher e seus familiares a permanecerem em casa, procedimento possível nos casos em que a classificação de Manchester for verde ou azul - em todos os outros casos a mulher é encaminhada à maternidade de referência. A consulta da enfermeira da ambulância só termina quando a gestante é acolhida na referência, garantindo a continuidade da assistência humanizada de enfermagem.

Vale ressaltar que em novembro de 2024 iniciou-se o processo de informatização do serviço. Assim, os registros ora manuscritos atualmente são realizados em um aplicativo específico acessado por um dispositivo móvel, armazenados em nuvem, e enviados a unidade assistencial que atenderá a mulher. A proposta é que a classificação realizada pela enfermeira da ambulância seja imediatamente comunicada à equipe da maternidade, para que se planejem os recursos necessários à assistência.

Em relação aos indicadores do serviço, o levantamento de dados de uma das equipes mostrou que foram feitos 692 chamados durante quatro meses de 2013^(5)^, e que de 2012 até 2023 as 12 ACC responderam a um total de 174.476 solicitações (média 14.549/ano). O levantamento recente dos dados do portal de transparência da Prefeitura do Rio de Janeiro (disponíveis no site: https://osinfo.prefeitura.rio/) mostram ainda que nos anos de 2023 e 2024 foram realizadas 24.730 chamados para a ambulância, dos quais apenas 1.395 não foram atendidos devido à cancelamento da central ou à saída da mulher da unidade básica ou da residência antes da chegada da ambulância, totalizando 94,4% de chamados efetivamente assistidos. Destes, 100% das fichas de atendimento foram completamente preenchidas e assinadas pelas enfermeiras, e 99,8% das usuárias ficou satisfeita ou muito satisfeita com o serviço. Neste mesmo período, praticamente a metade dos atendimentos (48,9%) demoraram menos de 30 minutos desde o disparo até a chegada na usuária, valor expressivo, considerando as dimensões do município (1.200,33 Km^2^), a sua complexidade geográfica e o número de ambulâncias.

A assistência à mulher na ACC é norteada pelos conceitos da autonomia, da proteção aos direitos da mulher, da resolutividade e do cuidado compartilhado que norteiam o modelo humanizado. Portanto, considera-se que nesse cenário de cuidado, o acolhimento e a classificação de risco levam à tomada de decisões da enfermeira a partir de uma escuta qualificada, associada ao julgamento clínico embasado em protocolo fundamentado cientificamente.

## DISCUSSÃO

A mortalidade materna no Brasil permanece em níveis elevados e são desenvolvidas políticas, programas e ações que visam reduzir estes números. No Rio de Janeiro foi criado o CC, cujo módulo logístico criou uma ambulância com características próprias. Aqui, buscou-se relatar a assistência prestada pelas enfermeiras na ACC, comparar com ambulâncias de atendimentos a gestantes de outros locais e discutir o serviço conectando suas ações com o Modelo dos Três Atrasos, destacando a forma como podem contribuir para a redução da mortalidade.

Sabe-se que os fatores socioeconômicos, culturais e a percepção do serviço são a chave para entender o primeiro atraso. Apesar de não ser possível alterar diretamente as questões socioeconômicas, reforçar o vínculo com o pré-natal e com a maternidade de referência, bem como apresentar e reforçar a importância do uso da ambulância, são atitudes que impactam nos fatores culturais e na percepção do serviço. Outros modelos de ambulância de gestante de fora do país não realizam atendimentos domiciliares, mas funcionam como um meio de transporte seguro de uma unidade de saúde para outra^(7,8)^. Diferentemente, a ACC vai ao domicílio, realiza um cuidado rápido, humanizado e muito bem avaliado pelas usuárias que, muitas vezes, atende às demandas da mulher e acompanhantes na própria residência ao fazer orientações e sanar dúvidas. Além disso, os profissionais são treinados e cobrados rotineiramente para que seja prestado serviço de qualidade, o que deve repercutir nas usuárias, resultando no aumento da confiança no sistema de saúde e na busca dos serviços em tempo oportuno.

As ações da ACC, cuja intenção é reduzir o segundo atraso, também envolvem outros módulos do CC. No município, toda mulher, exceto nos casos em que ela é referenciada ao pré-natal de alto risco, é informada pela equipe da unidade básica sobre a maternidade de referência, que é pensada pela gestão municipal com base na proximidade entre a residência e o hospital. Além disso, a quantidade de ambulâncias e o local de onde são disparadas foram pensados com o objetivo de reforçar a assistência nos locais com maior vazio assistencial e dificuldade de acesso. Todas estas ações facilitam a identificação e a chegada ao serviço, pois evitam superlotação e facilitam/fornecem meios de transporte^(2)^.

A experiência em países africanos mostrou diminuição na taxa de mortalidade materna com a implementação de ambulâncias tripuladas por enfermeiras ou parteiras que receberam treinamentos específicos para atenderem urgências e emergências obstétricas^(7,8)^. No caso da ACC, o número do telefone é fornecido à mulher no pré-natal e o atendimento fica à distância de um telefonema (ferramenta barata e amplamente disponível à população do município). Após avaliação pelo profissional da ambulância, caso seja necessário, o encaminhamento ao serviço adequado é feito gratuitamente e de forma segura para a unidade de referência. Portanto, a ACC amplia as atividades prestadas pelas ambulâncias africanas e mostra-se um serviço seguro e que reduz custos da mulher, ratificando que é uma estratégia eficaz contra o segundo atraso^(2)^.

O terceiro atraso refere-se a alguns aspectos da qualidade da assistência prestada, e perpassa a tomada de decisão do profissional. A decisão é mais acertada quanto mais bem treinado for o profissional. Neste sentido, as ambulâncias tripuladas por parteiras e enfermeiras treinadas da Gâmbia e de Burundi apresentam ótimos resultados^(7,8)^, enquanto enfermeiros especialistas em atendimento pré-hospitalar sentem-se assustados e apreensivos, pois o parto é um evento especial e raro que pode ter complicações que eles não sabem manejar^(9)^. Da mesma forma que nos modelos de ambulância africanos, a ACC é tripulada por enfermeiras treinadas, que fazem atividades de educação permanente continuamente, a fim de reduzir falhas de decisão e oferecer cuidado adequado já na residência.

A ampliação e a assertividade na comunicação são outro aspecto da ACC que pretende reduzir o terceiro atraso. Esta comunicação direta foi efetiva em Burundi, África, onde a equipe de saúde ao receber informações sobre o caso previamente podia planejar a assistência adequada^(8)^. A ação na ACC que se assemelha é a implementação do uso do tablet com aplicativo de comunicação direta entre a equipe da ambulância e da maternidade. No entanto, ela foi implementada recentemente, não tendo ainda seu impacto mensurado. Além da comunicação precoce, o uso da classificação de risco e do MEOWS também são ferramentas que sistematizam a assistência e sinalizam para todos os envolvidos os atendimentos prioritários e aqueles que necessitam de ação precoce.

Vale destacar que um serviço de ambulância de Botucatu, São Paulo, tripulado por profissionais que não são especialistas na atenção obstétrica, atendeu em 2012 um total de 358 mulheres com agravos obstétricos e avaliou a pertinência dos atendimentos com base na classificação de risco. Para os autores, apenas 24,4% dos atendimentos foram considerados pertinentes (classificados nas cores amarela, laranja ou vermelha)^(10)^. Na ACC todos os atendimentos, independente da classificação, são considerados pertinentes, pois são mais uma oportunidade de orientar, promover acessibilidade e a qualidade da assistência à mulher. Além disso, o número de atendimentos do programa revela que se trata de um serviço grande com proporções muito superiores aos observados em outros locais no Brasil^(10)^ e no exterior^(7-9)^. Estes dados refletem a importância do serviço, seu tamanho e o impacto que tem na saúde materna e neonatal.

A principal limitação do estudo é que, por se tratar de um relato de experiência com discussão teórica sobre os atrasos, não foram trazidos dados que mostrem os impactos da implementação da ambulância na redução dos três atrasos. Ainda assim, acredita-se que a ACC promova a redução da peregrinação e das filas nas maternidades, a transferência responsável da atenção básica ou das maternidades para a unidade de referência e a ampliação do acesso à saúde (dentro e fora das instituições para todas as mulheres do município). Além disso, a assistência oportuna, o transporte seguro, o uso da classificação de risco e do MEOWS, além dos treinamentos continuamente oferecidos à equipe de enfermeiras e condutores associado ao cuidado humanizado, são práticas que tornam a ACC não apenas bem-sucedida em seus objetivos mas também um serviço de atendimento pré-hospitalar para gestantes e puérperas baseado na humanização da assistência ainda não descrito na literatura, que pode ser responsável pela redução da mortalidade materna e neonatal por prevenir os três atrasos, bem como servir de modelo para outros locais.

## CONSIDERAÇÕES FINAIS

É possível notar que a assistência desenvolvida pelas enfermeiras do módulo transporte do CC é baseada no modelo humanizado e tem ações que dialogam diretamente com o Modelo dos Três Atrasos. Conclui-se que a atuação da enfermeira da ambulância para evitar o primeiro atraso depende, em parte, da divulgação do serviço pela equipe do pré-natal, adicionalmente à qualidade do serviço prestado, principalmente nos casos em que a mulher permanece em casa. A disponibilidade do serviço de ambulância, por si só, evita o segundo atraso. E o fato de a ambulância ser tripulada por enfermeira altamente treinada para atenção obstétrica é um fator que inibe o terceiro atraso. De forma conexa, a classificação de risco com o uso do MEOWS, e a comunicação direta entre a ambulância e a emergência da maternidade também são ferramentas que impedem o último atraso.

Portanto, as ações desenvolvidas de forma sistematizada visam evitar as demoras no acesso à unidade adequada às necessidades da mulher e ao cuidado oportuno. Diante da ausência de experiência similar descrita na literatura, o modelo de atendimento pré-hospitalar obstétrico desenvolvido pelo CC pode ser considerado pioneiro na iniciativa de qualificar a assistência materna e neonatal, e consequentemente reduzir a mortalidade materna.

## Data Availability

Os dados de pesquisa estão disponíveis no corpo do artigo.
